# Editorial: Viral vector-based gene therapy in neurological disease: The future is now

**DOI:** 10.3389/fneur.2023.1153681

**Published:** 2023-02-14

**Authors:** Jared B. Smith, Laura Cancedda, Sergiusz Jozwiak, Andrew C. Mercer

**Affiliations:** ^1^Research and Early Development, REGENXBIO Inc., Rockville, MD, United States; ^2^Brain Development and Disorders Lab, Istituto Italiano di Tecnologia, Genoa, Italy; ^3^Research Department, The Children's Memorial Health Institute (IPCZD), Warsaw, Poland

**Keywords:** adeno-associated virus (AAV), gene therapy, promoter, spinal muscular atrophy (SMA), viral vector, antibody, gene replacement

In this special issue of Frontiers in Neurology, we have focused on highlighting the advances being made using viral vectors as a medium for gene therapies to address diseases of the central nervous system (CNS). This emerging area of medicine, particularly in the domain of adeno-associated virus (AAV) vectors, has seen a dramatic increase in research, clinical trials, and more recently approved therapeutics over the last decade. However, this technology is still nascent, with more research necessary to fully understand both its potential and its pitfalls. This is particularly true for its use in the CNS, where AAVs have the potential to be truly revolutionary medicines for diseases with significant unmet medical needs that have not been addressable *via* earlier medical technology. The use of AAVs can be achieved *via* numerous routes of administration in order to target focused regions of the brain or broad expression throughout the CNS to produce a diverse set of transgene payloads, including proteins for gene replacement, micro-RNAs for gene knockdown, or antibody expression, as reviewed by Marino and Holt in this special issue. Across the remaining articles in this issue, two major themes emerge: a focus on promoters in controlling expression to occur selectively in particular cell types within the CNS; and insights into ongoing clinical uses of AAVs in spinal muscular atrophy.

## Promoters for cell-type-specific expression

A significant advantage of AAV approaches to gene therapy is the ability to restrict transgene expression to specific cellular subtypes of interest. A wealth of recent papers has revealed novel promoter/enhancer sequences that can be used to target subtypes of neurons in the CNS, with a particular focus on GABAergic neurons (1–4). As discussed in the review by Duba-Kiss et al. in this special issue, this focus on GABAergic subtypes is largely motivated by a plethora of evidence linking these cells to various neurological and neuropsychiatric disorders. Importantly, the authors also point out that the effectiveness of these promoters can vary widely across brain regions, across ages (neonatal vs. adult), and across species (see their Table 1 for a concise summary of these factors across recent studies).

**Figure 1:**
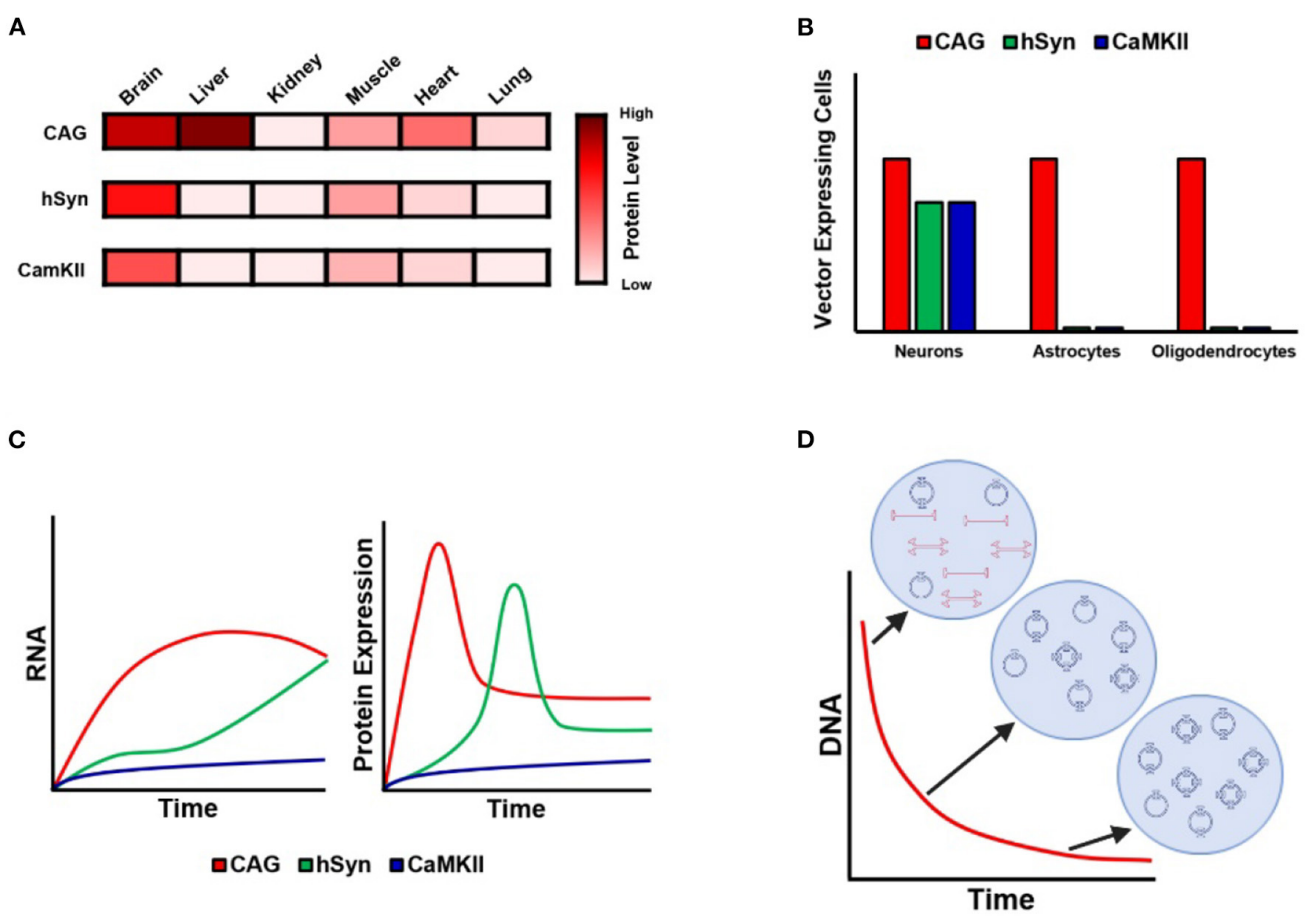
Kinetics of AAV expression with ubiquitous and neuron-specific promoters. **(A)** Illustration of GFP protein expression in different organs following administration of PHP.eB employing CAG, hSyn, and CamKII promoters. Note that CAG evinces strong expression in the liver and heart, whereas hSyn and CamKII are associated with almost no expression outside the brain, with the exception of weak expression in skeletal muscle. Data from Finneran et al.. **(B)** Cell-type-specific expression of GFP under CAG, hSyn, and CamKII following AAV9 intraparenchymal injection into the striatum. Note that CAG is associated with expression in neurons, astrocytes, and oligodendrocytes, whereas expression is restricted to neurons in the case of hSyn and CamKII. Data from Hollidge et al.. **(C)** Diagram of the 6-month time-course of RNA and protein expression under CAG, hSyn, and CamKII following AAV9 intraparenchymal injection into the striatum. Note the different kinetic profile of expression for each promoter. Data from Hollidge et al.. **(D)** Diagram of the 3-week time-course of vector DNA following AAV9 intraparenchymal injection into the striatum. Note that the total amount of vector DNA decreases continuously until stabilizing at 3 weeks, during which time the vector DNA forms stable circular episomes and concatemers of up to five vector copies per episome. Data from Hollidge et al..

Also in this issue, Finneran et al. deftly demonstrate that following intravenous administration of PHP.eB, a vector that crosses the blood–brain barrier, the vector achieves widespread CNS transduction. Importantly, the use of neuronal-specific promoters (hSyn1 and CaMKIIa) largely blocked expression in peripheral organs (the liver, heart, etc.), as the authors observed when employing CAG, a ubiquitous promoter (see Figure 1A). Hollidge et al. also compared these same promoters (CAG, hSyn, and CaMKIIa) following direct intraparenchymal delivery to the striatum; they confirmed that hSyn and CaMKIIa restrict expression to neurons only, whereas CAG elicits expression in neurons, astrocytes, and oligodendrocytes (Figure 1B). However, an important result from Hollidge et al. is that these different promoters also exhibited very different expression profiles over a 6-month period (Figure 1C). Interestingly, RNA and protein expression were found to diverge at later time points: specifically, a time point was identified at which RNA was still increasing, but protein expression levels decreased. These findings raise the need for further research to characterize the mechanisms affecting AAV transcription and translation over time and warrant consideration in the implementation of long-term studies. Finally, these authors also demonstrate that vector DNA undergoes continuous processing over the first 3 months after injection (Figure 1D): in particular, over the first 3 weeks after injection, the total amount of vector DNA diminishes as the remaining vector DNA assembles into stable circular episomes that concatenate with up to 5 copies per circular episome.

## Updates on clinical uses of AAV in the CNS

Beyond basic research into AAV biology and transgene cassette technology, this issue includes two updates on the challenges of AAV therapeutics from a clinical perspective. In Kotulska, Fattal-Valevski, et al. the authors review a currently available AAV therapeutic approved for clinical practice in patients with spinal muscular atrophy (SMA). Until now, most patients with acute forms of SMA have had a fatal prognosis before the end of the second year of life, due to general muscle hypotonia resulting in respiratory insufficiency. A major recent advance has occurred in the form of the introduction of *SMN1* gene therapy, specifically *via* onasemnogene abeparvovec, which employs a self-complementary adeno-associated virus 9 (scAAV9) vector to deliver a healthy copy of the *SMN1* gene (5). Phase 1 and phase 3 clinical trials have shown that a single administration of onasemnogene abeparvovec results in improvement of motor functions in the majority of infants with SMA. Kotulska, Fattal-Valevski, et al. review currently ongoing phase 3 clinical trials in SMA1 and SMA2 patients, as well as pre-symptomatic infants.

In the other article by Kotulska, Jozwiak, et al., the authors identify an important issue regarding gene therapy in neonates with SMA. Recently introduced in some countries, newborn screening programs allow very early use of gene therapy. However, the necessity of steroid regimens is problematic with the administration of neonatal live vaccines, especially the tuberculosis vaccine. The timing of gene therapy in such patients has not yet been addressed in the existing international guidelines. In the article, the authors present the first recommendations from the Polish Vaccinology Association for gene therapy administration in newborns who have received live vaccination against tuberculosis, which is currently used in clinical practice in Poland.

## Author contributions

All authors listed have made a substantial, direct, and intellectual contribution to the work and approved it for publication.
